# Consequences of Infectious Disease Outbreaks in Ardabil (1925–1941): A Historical Analysis

**DOI:** 10.34172/aim.31949

**Published:** 2024-12-01

**Authors:** Zahra Aghabeiglooei, Jamal Rezaei Orimi, Somaiyeh Marghoub Khajeh, Morteza Mojahedi, Farzaneh Ghaffari

**Affiliations:** ^1^Traditional Medicine Clinical Trial Research Center, Shahed University, Tehran, Iran; ^2^Department of Traditional Medicine, School of Persian Medicine, Shahed University, Tehran, Iran; ^3^Pre-Hospital Emergency Medical Services and Disaster Management Center, Mazandaran University of Medical Sciences, Sari, Iran; ^4^Department of Persian Medicine, Faculty of Medicine, Mazandaran University of Medical Sciences, Sari, Iran; ^5^Department of History of Medicine, Faculty of Traditional Medicine, Student Research Committee, Tabriz University of Medical Sciences, Tabriz, Iran; ^6^Traditional Medicine and History of Medical Sciences Research Center, Health Research Institute, Babol University of Medical Sciences, Babol, Iran; ^7^Mizaj Health Research Institute (MHRI), Tehran, Iran; ^8^Medical Ethics and Law Research Center, Shahid Beheshti University of Medical Sciences, Tehran, Iran; ^9^School of Traditional Medicine, Shahid Beheshti University of Medical Sciences, Tehran, Iran

**Keywords:** Ardabil, First Pahlavi period, Health, History of medicine, Infectious diseases

## Abstract

Infectious diseases were one of the most important public health problems in Ardabil during the first Pahlavi period (1925-1941 AD). These diseases caused the illness and death of many people. The purpose of this study is to investigate the factors and consequences of the spread of infectious diseases in Ardabil during the first Pahlavi period. The research method is descriptive and historical-retrospective, which tries to answer the research questions using documentary and library sources. The findings show that malaria, trachoma, smallpox, tuberculosis, rinderpest, venereal diseases, and diphtheria were among the most common infectious diseases. Poverty, illiteracy, poor public health, lack of medical facilities, and the geographical location were the main factors in the spread of these diseases. Considering the critical nature of the issue, the first Pahlavi government tried to control and prevent diseases by carrying out measures such as public education, establishing health and treatment centers and reforming their structure, sending doctors and distributing medicine, and implementing quarantine and vaccination. The results showed that the efforts of the first Pahlavi government in fighting infectious diseases in Ardabil were relatively successful and reduced the prevalence of some diseases.

## Introduction

 Ardabil is one of the northwestern provinces of Iran, located on the southern slopes of Savalan mountain and next to the Qareh Su River.^1^ This province is bordered by the Republic of Azerbaijan to the north, Gilan province to the east, Zanjan province to the south, and East Azerbaijan province to the west.^2^ Ardabil is one of the ancient and historical cities of Iran, which was the center of Azerbaijan before and after Islam.^3-5^

 Due to its unique geographic location, Ardabil has historically been susceptible to outbreaks of infectious diseases. During the Safavid era, the plague, one of the deadliest diseases, repeatedly ravaged Ardabil and other parts of Iran. The plague of 1573, which killed approximately 30 000 people in Ardabil, was one of the most devastating outbreaks.^6^ In 1624, a deadly contagious disease spread throughout the regions of Ardabil, Khalkhal, and Sarab, claiming the lives of nearly 100 000 people.^7,8^ The prevalence of infectious diseases continued in Ardabil and other regions during the Qajar era. In 1832, cholera and plague spread in Ardabil.^9^

 Based on this, one of the most important public health challenges in Ardabil during the first Pahlavi period (1925-1941 AD) was the spread of contagious infectious diseases.^10^ Diseases such as cholera, plague, smallpox, and typhoid, not only threatened people’s health but also had profound effects on social structures. Among the main consequences of the spread of infectious diseases, we can mention the increase in the death rate, increase in treatment costs, decrease in production and productivity, and damage to economic development.^11^

 Despite the importance of the issue, there is no comprehensive information about the spread of infectious diseases in Ardabil during the first Pahlavi period, and the available historical sources provide scattered and incomplete information about this issue.^12-15^ Despite examining the health and treatment situation, these researches did not pay much attention to the prevalence, causes and consequences of diseases in Ardabil and only made a brief reference.

 Given the nature of the subject, the research methodology employed is a historical, descriptive, and retrospective study. By utilizing documentary and library sources, this research aims to answer research questions regarding infectious diseases in Ardabil. In this study, information was gathered and indexed based on research objectives using keywords such as “Ardabil, infectious diseases, contagious diseases, quarantine, and Health.” After organizing and analyzing the data, conclusions were drawn.

 Building upon previous research, this study seeks to examine the factors and consequences of infectious disease outbreaks in Ardabil from a historical perspective.

## Hygienic Condition of Ardabil During the Beginning of the First Pahlavi Era

 At the beginning of the first Pahlavi period, the health and treatment situation in Ardabil was poor. Lack of medical and health facilities plagued the people of this region and the death rate from various diseases was terribly high. Diseases such as smallpox, plague, malaria and trachoma killed many people, especially children. For example, in a report from 1925, the head of health department of Ardabil describes the health and treatment situation of this city as “deplorable”. According to him, Ardabil not only lacked proper administrative structures in this field, but it was also deprived of an equipped hospital and aid post.^16,17^

 In another report from 1926, it is mentioned that there is a severe shortage of medicine and medical equipment in Ardabil. During this period, Ardabil had only one pharmacy and a basic hospital which was more like a convalescent home. The severe lack of medical equipment and facilities, along with the absence of any health supervision, made the situation worse. Ardabili doctors, despite their efforts, used traditional and old methods to treat patients, and there were no educated doctors in this region.^18-20^

## Outbreak of Infectious Diseases in Ardabil During the First Pahlavi Era

 According to the available documents, during the first Pahlavi period, malaria, trachoma, smallpox, tuberculosis, sexually transmitted diseases, plague and diphtheria were among the most common diseases in this period.^5,21^ Table 1 shows the prevalence of different types of infectious diseases in Ardabil during the mentioned period.

**Table 1:** Outbreak of Infectious Diseases in Ardabil During the First Pahlavi Era.

**No.**	**Content**	**Year**	**Reference**
1	Rinderpest outbreak in Ardabil	1925	^22^
2	Small pox outbreak in Ardabil different era	1927	^20^
3	Livestock outbreak in Anbaran village of Namin	1928	^23^
4	Diphtheria outbreak in Namin, Vilkij of Ardabil	1930	^24^
5	Plug outbreak in villages of Tazeh kand and DashBulagh in Ardabil	1932	^25^
6	Trachoma outbreak in Ardabil and Astara’s schools	1935	^26^
7	infectious disease outbreak in Ardabil and Germi	1935	^27^
8	livestock diseases outbreak in Sanjabad village of Khalkhal	1935	^23^
9	Malaria outbreak in Astara and Bileh savar	1936	^28^
10	Malaria outbreak in Ardabil’ s schools	1937	^29^
11	Malaria outbreak in Astara and Namin	1937	^28^
12	Infectious disease outbreak like small pox in Namin	1937	^30^
13	Influenza outbreak in Bileh Savar’ s schools	1937	^31^
14	Syphilis outbreak in Ardabil	1937	^32^
15	Infectious diseases outbreak in Bileh Savar, Germi, and Meshginshahr	1940	^33^
16	Prevalence of malaria, small pox, diphtheria, and syphilis in Meshginshahr and Sarab	1940	^34^
17	Smallpox outbreak in Khalkhal	1940	^35^
18	Rubella outbreak in Khalkhal villages	1940	^36^
19	Infectious diseases outbreak like malaria, influenza, and rubella in Khalkhal	1941	^37^

## Malaria

**Figure 1:**
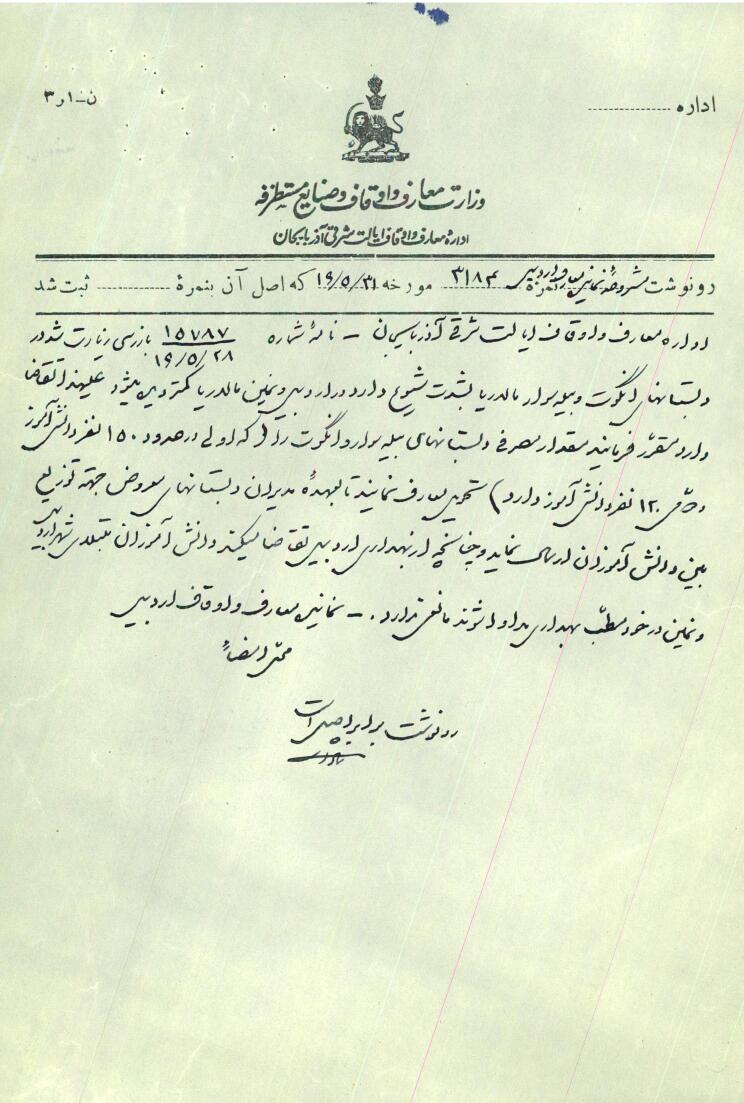
A Letter from the Ardabil head of the Department of Education and Endowment to the Azerbaijan Department of Education and Endowment in 1937 with regards to the malaria outbreak in Bileh Savar schools.^28^

 Malaria was a prevalent disease in Iran during the first Pahlavi era, primarily affecting the forest areas of Ardabil.^38^ Passengers traveling to Gilan were the primary carriers, posing a threat to people’s health and lives.^5^ In 1925, a research group under Dr. Amidzadeh found that around 53% of people in the south strands of the Aras River died due to malaria.^39^ Malaria also affected other areas of Ardabil, with Astara and Bileh Savar being designated as malarial districts in 1936.^28^ In 1937, schools in Ardabil were not safe from malaria’s impact, with students from various cities in the province suffering from the disease^29^ (Figure 1).

## Trachoma

 Trachoma, an inflammatory eye disease caused by *Chlamydia trachomatis* bacterial infection, was known as one of the main causes of blindness in Ardabil and surrounding areas in the past. This disease, which is transmitted through direct contact with the infected secretions of an infected person, spreads rapidly in poor sanitary conditions, especially in deprived areas. One of the primitive documentary reports about trachoma in Ardabil is related to the visit of Dr. Mohammad Ghasem Mirdamadi, who was a member of the Institut Pasteur, to the DashBulagh village in 1932. During this travel, while examining villagers, he observed some evidence of this disease and warned against the dangerous consequences of it, especially blindness. The next report about trachoma was presented by Dr. Shams Hakemi, a doctor working in the hospital of this city, in 1935 regarding Ardabil schools.^25,26^

## Smallpox

 Smallpox was one of the most prevalent infectious diseases in Ardabil. Evidence and many reports imply that the annual outbreak of smallpox with different intensities in different parts of Ardabil presented these diseases as one of the predominant cases in this district^40^ (Table 1). In 1927, smallpox outbreaks in Ardabil led to child deaths. In 1937, the prevalence of smallpox in Meshginshahr took the lives of some people. Following this incident, the General Department of Health detached doctors and medicine to these areas to confront this disease.^41^

## Venereal Diseases

 Venereal diseases such as gonorrhea and syphilis were instances of common diseases among people from Ardabil. Hakim Haroon, a Jewish doctor from Ardabil, mentions in his diary about treating patients who had syphilis in his clinic. This report is representative of the existence of this disease among the citizenry and their doctors’ visits for treatment.^21^ In some sections, disregard for ethical issues and the spread of promiscuity caused an increase in venereal diseases in Ardabil.^42^ Also, lack of knowledge about ways of contracting venereal disease were effective causes of their spread. In 1940, control of syphilis was poor in Meshginshar and Sarab.^34^

## Diphtheria

 Diphtheria is a contagious and life-threatening disease historically regarded as a major cause of mortality, particularly in children.^43^ In 1930, diphtheria spread among the people of Namin (one of the cities of Ardabil). This disease killed many people, especially children, and created panic and anxiety among the people. The people of the region, who were upset with the spread of diphtheria and the deaths caused by it, wrote a letter to the State Health Department, requesting for a doctor to be sent to their region.^24^

## Tuberculosis

**Figure 2:**
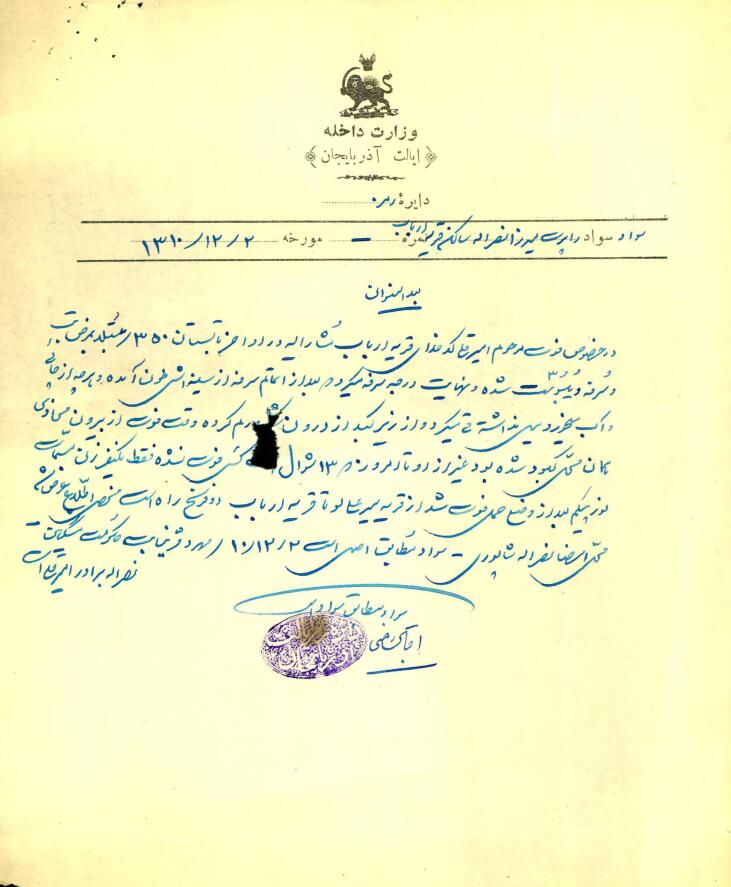
Mirza Nasrullah’s Report, Inhabitant of Arbab Village, with Regards to the Disease of the Head Man in This Village^25^

 Tuberculosis (TB) is a contagious respiratory illness characterized by the infection of the lymph nodes and lungs.^44^ During the late Qajar era (1796-1925) and the early Pahlavi era,^45^ TB was particularly prevalent in the northeastern province of Azerbaijan (Ardabil) and along the Caspian Sea coast in northern Iran.^46^ This disease was common among carpet weavers in Ardabil.^5^ Unsuitable working conditions, poor ventilation, and inaccessibility of hygienic facilities put the carpet weavers at risk of contracting this disease. In 1931, one of the residents of “Meshginshahr” contracted tuberculosis and died suddenly. Mirza Nasrulleh, a resident of Arbab village, says: “he had a fever, caught constipation, and was caught severely, and his lungs were filled with blood. He swelled intra-abdominally, right beneath the liver”^25^ (Figure 2).

## Plague

 The plague was one of the numerous contagious illnesses that were said to be deadly in Ardabil. When the epidemic reached Ardabil in 1932, the general consul of Russia in Tabriz reported the deteriorating situation. The outbreak of plague in Tazeh Kand and Dashbulagh villages, which claimed all lives of the villagers, was reported to higher authorities. The accuracy of the story was confirmed by an Armenian doctor and Russia’s general counsel during their field study visit to Ardabil. The research demonstrated how local authorities’ incapacity and ignorance prevented them from taking the necessary preventative and curative measures, which accelerated the disease’s spread and raised the death toll.^25^

## The Flu

**Figure 3:**
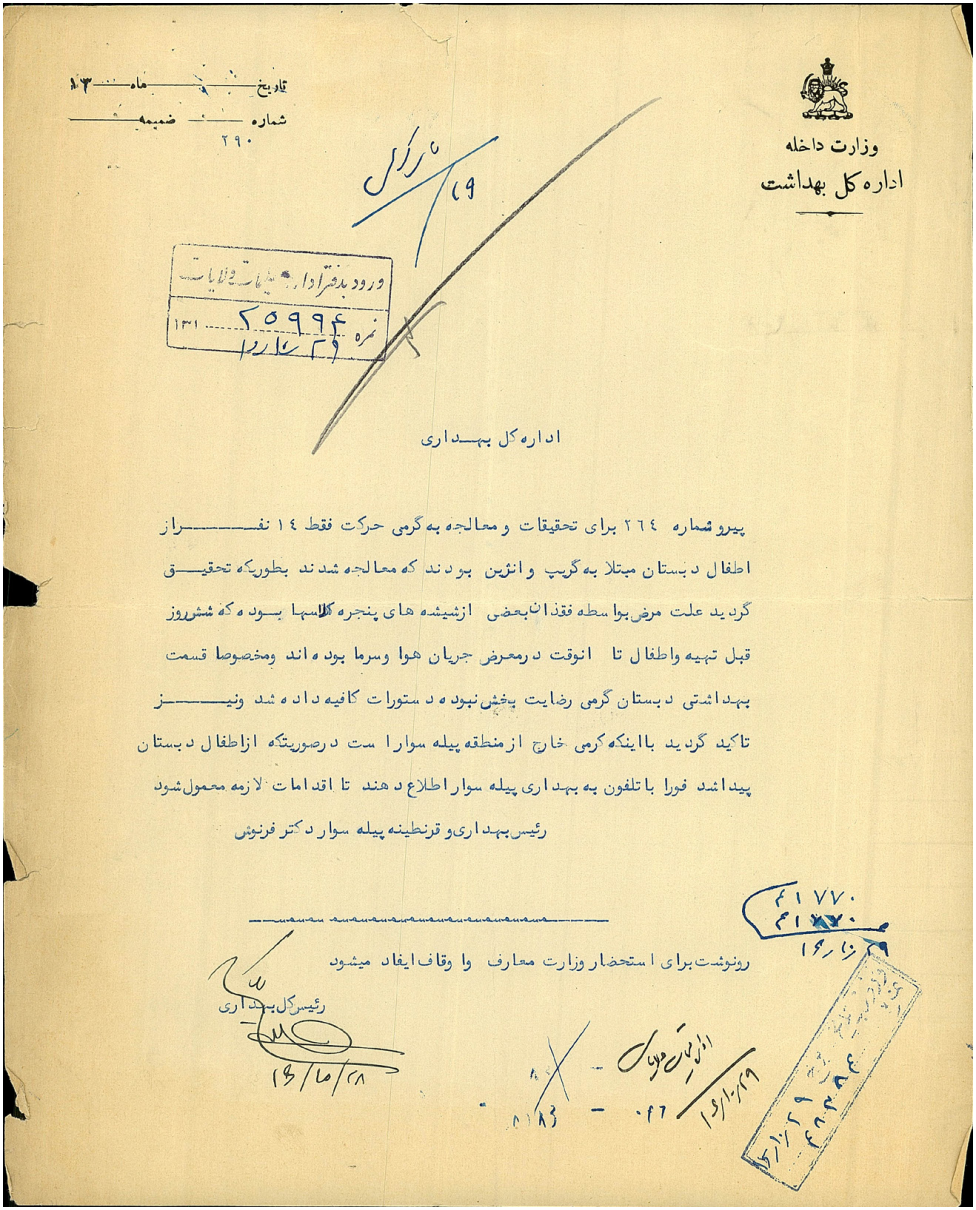
Head of Dispensary and Quarantine of Bileh Savar, Dr. Farnoosh’s Letter to the Department of Dispensary in 1937 with Regards to the Health Condition of Bileh Savar School and the Flu Outbreak^31^

 Influenza was one of the most common diseases in Iran in the late Qajar and early Pahlavi periods. The northwest region of Iran is where the flu primarily enters the country.^47^ There are numerous reports about the outbreak of the flu in Ardabil during the studied period.^27,30^ One of these cases was an outbreak of the flu in 1937 among students at Germy Elementary School. After the report of the flu outbreak in the elementary school of Germy, Dr. Farnoosh, Bileh Savar head of the dispensary, was sent there to treat patients and analyze the causes of the disease outbreak. After looking over the kids, Dr. Farnoosh determined that fourteen of them had the flu^31^ (Figure 3).

## Backgrounds and Causes of Infectious Diseases in Ardabil During the Pahlavi Era

 Poor sanitary conditions in urban and rural areas were the main causes of the transmission of infectious diseases. Lack of access to healthy water caused transmission of infectious diseases.^27,48^ Reports and correspondences related to public hygiene represented that pollution of passages, drinking water, and subsequent diseases were crucial problems that involved municipalities and other relevant institutions in Ardabil.^49-51^

 Economic and social conditions had an important role in the occurrence of these diseases. Many of the people barely observed any individual and environmental hygiene measures due to poverty and inability to afford their substantial needs. On the other hand, changes in social life style, population increase, and migrations of people to cities were the causes that helped outspreading infectious.^52^

 The geographical location of Ardabil especially Astara city was one of the important causes of infectious diseases outbreak.^53^ Astara as a border city and the main passage of merchants and traders was prone to transmission of infectious diseases.^41,54^

 The relocation of Shahsevan nomads was one of the key factors for threatening public health and hygiene. These relocations provided grounds for transmission of disease from one point to another and spread common diseases between humans and animals. Analyzing the available documents and reports shows the existence of infectious diseases and illnesses like malaria and diarrhea among Shahsevan tribes.^55,56^

## Government Actions in Combating Infectious Diseases in Ardabil During the First Pahlavi Era

 At the beginning of this era, the fight against diseases started and some actions like completion of rules and regulations, establishment of health centers in towns, serious supervision to prevent outbreak of infectious diseases, establishment of the supreme council of health, cultivating, public and free vaccination, creating and reinforcing quarantine centers, and the establishment of Institut Pasteur took place.^57-60^ The government tried to prevent outbreak of diseases and, in case of outbreak, to be informed quickly and control them by compilation of rules and regulations. Documents related to rules of compulsory vaccination, the rule related to notification about infectious diseases, and monthly reports of municipal health department are some examples to be mentioned. Planning to fight against common diseases in Ardabil and surrounding cities started in this way.^61-63^

## Actions During Outbreak of Infectious Diseases

 The Iranian government took actions like medicine administration, vaccination, screening and quarantine to prevent infectious diseases in Ardabil. To fight against this disease, actions like the distribution of quinine pills and fighting against mosquito vectors took place.^28,29,64^ After the outbreak of malaria in schools in 1937 in Ardabil, an agent of the education and endowment department, presented approaches for treating the suffering students. One of the treatments was at the dispensary clinic. Ardabil and Namin’s school students who had malaria were sent to a dispensary clinic to receive treatment. In these centers, doctors and medical staff took necessary actions for students’ treatment with examination and diagnosis. Another approach to distributing medicine was via the schools principals.^28^

 The plan of public and free vaccination was approved in the beginning of Pahlavi era. To start the executive process of it, Azerbaijan’s state of health demanded the collaboration of Ardabil’s people with doctors and vaccinators with regards to the necessity of children’s vaccination and its implementing stages.^65,66^ In case of a smallpox outbreak, doctors and vaccinators were assigned and dealt with vaccination in this particular district.^34,35^

 One of the important actions of Ardabil dispensary was diagnosis and screening; for example, supervision and inspection of schools and examination of students.^67^ An example would be the visit of Dr. Mirza Asadollahkhan Sehati, who was a circulating doctor in the vicinity of Ardabil, to Astara and Ardabil schools during the outbreak of trachoma in 1935, and examining the students and asking for medicine.^68^

 Quarantines (temporary and permanent) as one of the key tools of controlling infectious diseases played an important role in preventing the outspread of diseases in Ardabil.^69^ Most quarantines were located in border areas like Astara and Bilehsavar, which were at higher risk of infectious disease outbreaks due to high travel and goods volume.^70,71^ During outbreaks in Russia and Iran, travelers were required to have a health certificate indicating vaccinations.^54,72^

## Modification of Sanitary and Therapeutic Structures

 During the first Pahlavi era, the Ardabil Department of Dispensary took significant actions in order to modify the sanitary system and treatment structure. These modifications were implemented in various fields, such as providing drinkable water, collecting trash, controlling nutrients, fighting against insects and animals carrying diseases, and public education.^73^ One of the key actions of the dispensary department was the provision of drinkable water to people. The dispensary department brought drinkable water to people by excavating deep holes and constructing piping networks.^73^

 Public education was one of the main aspects of the dispensary department’s programs to improve public health. This department educated people in many different individual and public hygienic fields by publishing health papers and notices, instructing teachers, and giving speeches. One of the important publications that was active in this field was the Jowdat publication. This publication made announcements in the field of hygiene and treatment, in addition to news and local reports.^74^

 During this period, the dispensary department supervised the hygienic condition of different crafts like bathhouses, inns, tea houses, cookery, and carpet workshops effectively. This supervision included the working environment, nutrition, and staff hygiene. In spite of the taken actions, there were still problems in the field of public hygiene.^75,76^

 During the first Pahlavi period, measures were taken to organize the treatment situation, including systematizing the affairs of doctors and pharmacies. The dispensary department tried to prevent the activity of illegal doctors by providing a list of legitimate doctors and pharmacists.^77-79^ Circulating health doctors were preferred and gradually, the number of educated doctors increased.^80^ The activity of drugstores was supervised more tightly during this period.^80,81^ New hospitals and health centers were founded in this district due to the attempts of the government, the municipality, and the Red Crescent.^82^ Astara Hospital was founded with the help of the Red Crescent and the Ministry of Interior in 1929.^83^ As time went on, the general department of the dispensary implemented basic modifications in Ardabil. Also, Ardabil municipality provided a great help for treating patients by founding a hospital. One of the key actions was creation of permanent circulating first aid stations by Ardabil municipality, which provided free service to patients all over the cities of the province like Khalkhal, Astara, Namin, and Bileh Savar.^16,30,84,85^

## Conclusion

 Investigations show that infectious disease outbreaks were common in Ardabil during the first Pahlavi era. Various factors such as deficient public health, inaccessibility of drinkable water, unfavorable economic and social conditions, inefficient government officials, shortage of medical facilities, geographical position of Ardabil, and nomads’ lifestyle played a role. The first Pahlavi government made efforts to fight against infectious diseases with actions such as educating and informing people, establishing health centers, providing doctors, implementing quarantine, and vaccination. These attempts were successful to some extent and caused a reduction in the outbreak of some diseases. Despite all these, the number of infectious diseases in Ardabil was considerably high. Due to the mentioned points, it can be concluded that infectious diseases were a serious challenge for public health in Ardabil. The first Pahlavi government’s actions to fight and control infectious diseases and improve the condition of public health in Ardabil were remarkable.
